# Microflow LC-MS Bottom-Up Proteomics Using 1.5 mm Internal Diameter Columns

**DOI:** 10.1021/acsomega.4c10591

**Published:** 2025-01-24

**Authors:** Siddharth Jadeja, Denis K. Naplekov, Mykyta R. Starovoit, Kateřina Plachká, Harald Ritchie, Jason Lawhorn, Hana Sklenářová, Juraj Lenčo

**Affiliations:** † Department of Analytical Chemistry, Faculty of Pharmacy in Hradec Králové, Charles University, Heyrovského 1203/8, 500 03 Hradec Králové, Czech Republic; ‡ Advanced Materials Technology, 3521 Silverside Road, Suite 1-K, Wilmington, Delaware 19810, United States

## Abstract

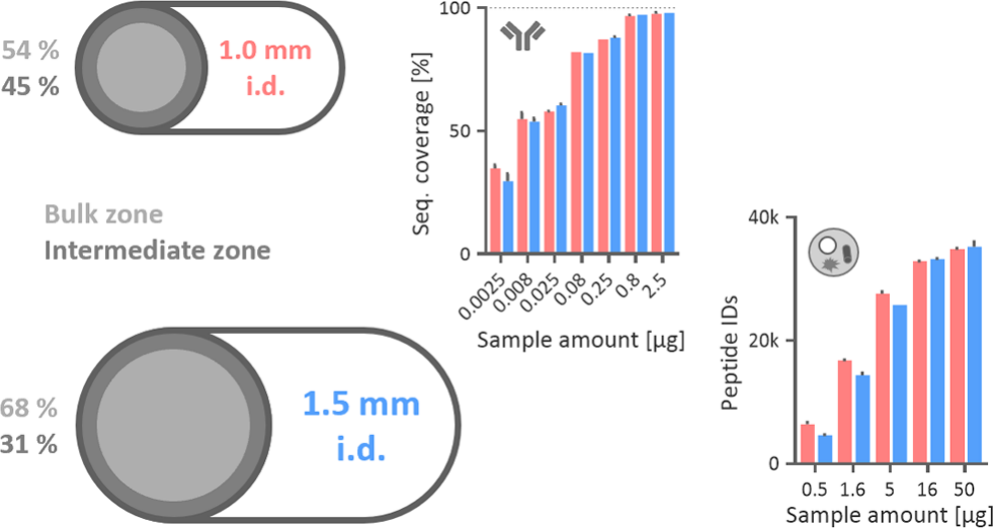

Microbore columns with a 1.0 mm inner diameter (i.d.) have gained popularity in microflow liquid chromatography–mass spectrometry (LC-MS) workflows for exploratory proteomics applications due to their high throughput, robustness, and reproducibility. However, obtaining highly efficient separation using these columns remains unachievable, primarily due to significant radial flow heterogeneity caused by uneven particle packing density across the column cross-section. In this study, we evaluated the integration of a 1.5 mm i.d. column, which offers greater packing uniformity and reduced radial flow dispersion, into a microflow LC-MS setup for bottom-up proteomics analysis. The performance of the 1.5 mm i.d. column was compared with that of the 1.0 mm i.d. column using protein samples of varying complexity. The results demonstrate that 1.5 mm i.d. columns provide superior chromatographic separation and better compatibility with conventional-flow LC systems, yielding higher reproducibility and comparable protein and peptide identifications to the 1.0 mm i.d. columns at higher sample amounts. These findings suggest that 1.5 mm i.d. columns could be a suitable alternative to 1.0 mm i.d. columns for microflow LC-MS/MS proteomic analysis, particularly in laboratories with only conventional-flow LC systems.

## Introduction

Chromatographic separation of peptides using capillary columns with an inner diameter (i.d.) of 100 μm or less coupled with a nanoflow liquid chromatographic system designed to operate at flow rates below 1 μL/min is considered the gold standard in proteomics research.^1−3^ However, advancements in highly sensitive mass spectrometry instruments and the necessity to analyze large numbers of samples have driven the demand for more robust, reproducible, and high throughput alternatives. In 2018, Lenčo et al. demonstrated that microbore columns with a 1.0 mm i.d. could effectively replace nanoflow capillary columns in standard proteomic applications, requiring only a roughly 5-fold increase in peptide sample input, as long as other parameters were well-optimized.^4^ The 1.0 mm i.d. columns provide a longer lifetime, high reproducibility, and robustness than capillary columns^1,5^ and can be seamlessly integrated into workflows using conventional-flow LC systems.^4^ This has enabled researchers to perform exploratory proteomics experiments using conventional-flow systems, which are commonly available in most analytical laboratories, eliminating the need for dedicated nanoflow chromatographic systems. However, they can also be adapted for nanoflow LC systems through suitable pump adjustments. The Vanquish Neo UHPLC system (Thermo Fisher Scientific) even eliminates the need for such modifications. The adoption of 1.0 mm i.d. columns brings a significant advancement in the field of proteomics, bridging the gap between nanoflow and conventional-flow chromatography. This transition proved to be crucial for enhancing throughput while providing sufficient sensitivity to proteomic workflows.

Although 1.0 mm i.d. columns outperform capillary nanoflow columns in separation performance, their efficiency is far from ideal. During packing, the column bed does not experience uniform pressure due to the presence of the fixed column wall. The pressure increases from the center toward the wall, leading to variations in particle bed density across the column’s cross-section. Reising et al. confirmed the existence of three coaxial zones in columns: (i) a thin, loosely and orderly packed region at the wall, with a thickness of approximately 1.5× the particle diameter; (ii) a thick, densely and randomly packed intermediate region around 130 μm thick; and (iii) a randomly packed bulk region.^6^ These zones, with different bed densities but without sharp delineation, cause uneven mobile phase flow and represent a major limitation in column efficiency. The degree of radial flow heterogeneity depends on the intermediate-to-bulk zone ratio. Gritti derived a stochastic model to predict this so-called trans-column eddy dispersion, linking column efficiency to the bed aspect ratio, i.e., the ratio of column i.d. to particle diameter i.d. The model predicts that 1.0 mm i.d. columns packed with sub-3 μm particles are among the least efficient, as each intermediate and wall zone occupy about half of the total column bed volume.^7^ Furthermore, as column volume decreases with reduced i.d., 1.0 mm i.d. columns become highly sensitive to extra-column band dispersion, making it critical to minimize postcolumn volumes to maintain their performance.^4,8−10^

Some researchers have adopted conventional-flow liquid chromatography–mass spectrometry (LC-MS) configurations to simplify and improve the robustness of their proteomic workflows. In columns with a 2.1 mm i.d., the intermediate zone occupies only about 25% of the column volume, resulting in a relatively uniform column bed. Moreover, analyte bands are predominantly localized in the central bulk region of the bed in these columns, with low radial dispersion into the intermediate and wall zones during axial migration. Gonzalez et al. identified 800 and 1,200 proteins from 40 μg of *Escherichia coli* and *Arabidopsis thaliana* protein digests, respectively, using a 2.1 mm i.d. analytical column at a 400 μL/min flow rate.^11^ Orsburn et al. introduced the Standard Flow Multiplexed Proteomics (SFloMPro) method for analyzing isobaric-tagged samples using a 2.1 mm × 150 mm column operated at 200 μL/min.^12^ This flow regime reduces the complexity and costs associated with nanoflow configurations while maintaining data quality, making it viable for high-throughput proteomics where sample availability is not a limiting factor. More recently, Ralser’s group employed 2.1 mm i.d. columns for ultrafast proteomics in a data-independent acquisition mode. Their study demonstrated that conventional-flow chromatography using 2.1 mm i.d. columns not only increases throughput and enhances peak capacity but also reduces sample carryover and improves electrospray ionization (ESI) performance.^13,14^ Despite these advantages, many practitioners exploiting conventional-flow LC-MS analyses still favor 1.0 mm i.d. columns over 2.1 mm i.d. columns in high-flow proteomic analyses due to the increased sensitivity achieved with narrower inner diameters.

To bridge the gap between 1.0 mm and 2.1 mm i.d. columns, Advanced Materials Technology has recently developed analytical columns with a 1.5 mm i.d. These columns offer superior chromatographic separation and better compatibility with conventional flow chromatographic systems compared to 1.0 mm i.d. columns.^15^ Their enhanced efficiency primarily stems from a more favorable bed aspect ratio when packed with sub-3 μm particles, coupled with reduced susceptibility to extra-column band dispersion. This configuration decreases the minimum reduced plate height significantly compared to 1.0 mm i.d. columns packed with the same particles. The 1.5 mm i.d. columns have shown some promise as replacements for 2.1 mm i.d. columns in peptide mapping of biopharmaceuticals.^16^ However, their suitability for bottom-up proteomic experiments remains to be determined. We hypothesize that the sharper peaks obtained as a result of better chromatographic separation of 1.5 mm i.d. column may deliver more precursor ions for fragmentation per unit time, improve the quality of MS2 spectra, and/or reduce the time required to obtain the MS2 spectra. These factors collectively may offset the naturally lower MS sensitivity of 1.5 mm i.d. columns due to the increased i.d., potentially improving proteome coverage compared to 1.0 mm i.d. columns, particularly in data-dependent acquisition (DDA) mode. In this study, we evaluated the 1.5 mm i.d. column and compared its performance with 1.0 mm i.d. columns in analyzing protein samples of varying complexity using a conventional high-flow chromatographic system.

## Experimental Section

### Reagents and Materials

Unless stated otherwise, chemicals and reagents were purchased from Sigma-Aldrich/Merck in the highest available grade. LC and LC-MS grade solvents and formic acid were purchased from Honeywell or Fisher. Alkylphenons standard mixture was from Agilent (RRLC Checkout Sample). iRT peptides were synthesized in a purity higher than 95% by Royobiotech (China). Unused leftovers of freshly reconstituted trastuzumab (Herceptin, Roche) were received from Multiscan Pharma, Czech Republic.

### Sample Preparation

Four different peptide samples with increasing complexity were used for the study: a mixture of 11 iRT peptides,^17^ a tryptic digest of an antibody biopharmaceutical trastuzumab, a tryptic digest of a live vaccine strain (LVS) of *Francisella tularensis*, and a tryptic digest of Jurkat cell proteins. The protein concentration in the lysates was determined using a bicinchoninic acid assay (Sigma-Aldrich). Sample preparation is described in detail in the Supporting Information (Note S1).

### Measurement of True Column Efficiency

The intrinsic height equivalents of theoretical plates (HETP) for all columns were measured using the procedure developed by Gritti and Guiochon.^18^ Measurements were performed at three linear velocities of the mobile phase near the optimum. The experiments were conducted using an Acquity UPLC I-Class system (Waters). The columns were connected to a UV detector via 350 mm capillaries with i.d. of 100, 75, and 50 μm. The peak variance was calculated from peak width at half height (*w*_h_).

### LC-MS Analyses

LC-MS analyses were conducted using a conventional-flow Vanquish Horizon UHPLC system hyphenated to a Q Exactive HF-X mass spectrometer (Thermo Fisher Scientific). All columns used in this study were 150 mm long and packed with 2.7 μm superficially porous Halo Peptide 160 Å ES-C_18_ particles (Advanced Materials Technology). The inner diameters of the columns were 1.0, 1.5, and 2.1 mm. Columns were operated at 55 °C and flow rates 51, 115, and 225 μL/min. A 150 mm long × 50 μm nanoViper capillary was used to connect the separation columns with 1.5 mm and 1.0 mm i.d. to the ESI source. The nanoViper capillary was not compatible with the ports of the 2.1 mm i.d. column and was therefore replaced with a 50 μm × 200 mm SecurityLink capillary (Phenomenex).

The ESI voltage was 3.5 kV for all three flow rates. The columns were connected to the HESI-II ion source with capillaries with an inner diameter of 50 μm to minimize the postcolumn peak broadening. The HESI-II settings recommended by the control software for each flow rate were used, as mentioned in Table S1. The MS1 and DDA settings for particular experiments were identical for each column diameter and are specified in Table S2.

The mobile phase was formed from component A (0.1% formic acid in water) and component B. For analyses of iRT peptides and tryptic digest of trastuzumab, for an accurate evaluation of chromatographic peaks, component B contained 80% acetonitrile, 19.9% water, and 0.1% formic acid, keeping the viscosity of both components closer to each other, leading to a smoother pressure profile. Component B contained acetonitrile with 0.1% formic acid for *F. tularensis* (LVS) and Jurkat cell digest analyses.

Unless otherwise stated, the samples were injected in duplicates to verify reliability and repeatability. Details on how the LC-MS data were evaluated are specified in the Supporting Information (Note S2). All LC-MS files were deposited in the ProteomeXchange repository with the identifier PXD057525.^19^

## Results and Discussion

### Packing Uniformity and Intrinsic Efficiency of Columns with Varied Inner Diameter

**Figure 1:**
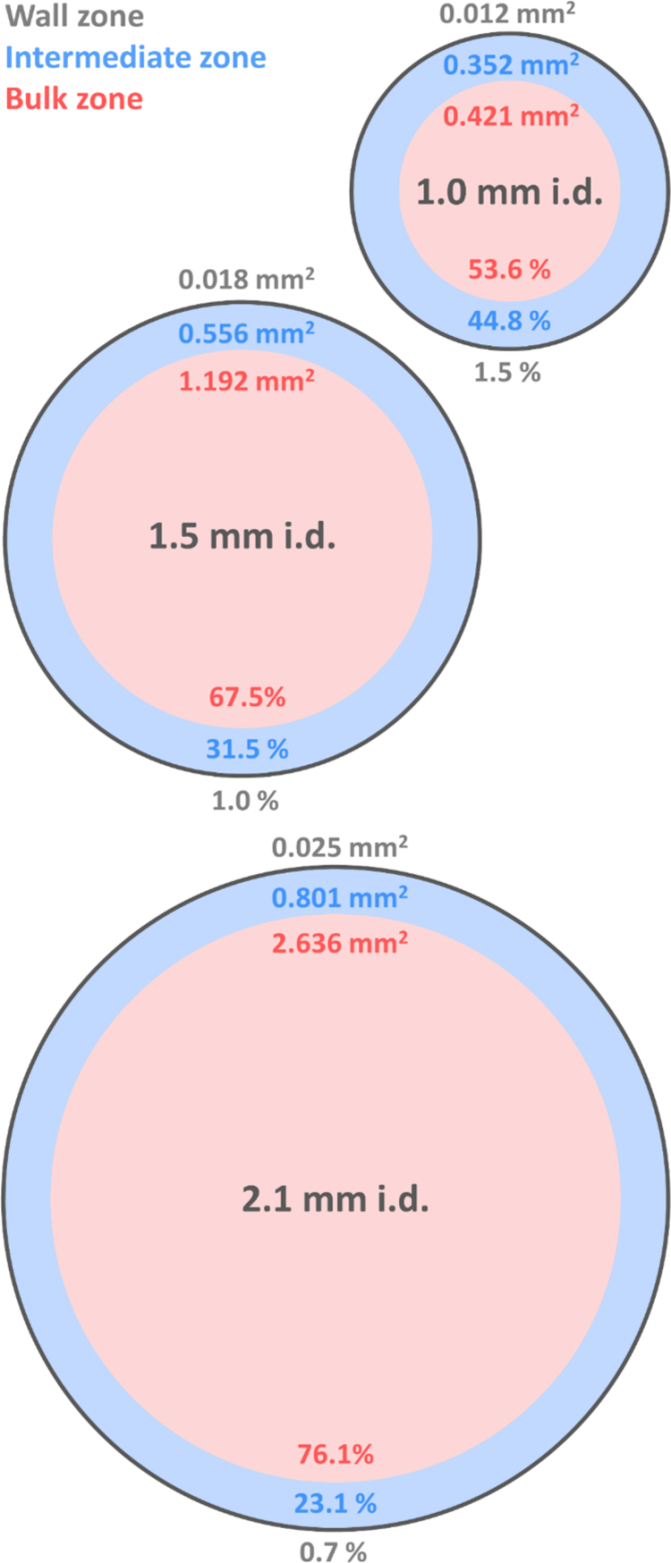
A schematic representation of three different radial zones of columns with 1.0, 1.5, and 2.1 mm inner diameters packed with 2.7 μm particles. Particles are loosely and orderly packed in the wall zone (gray). The particles of the intermediate zone (blue) are randomly and densely packed compared to the bulk zone (red). Based on the inner diameter of the columns, the two main zones occupy a different relative portion of the column cross-section. The calculations were done based on the stochastic model derived by Gritti.^7^

**Figure 2:**
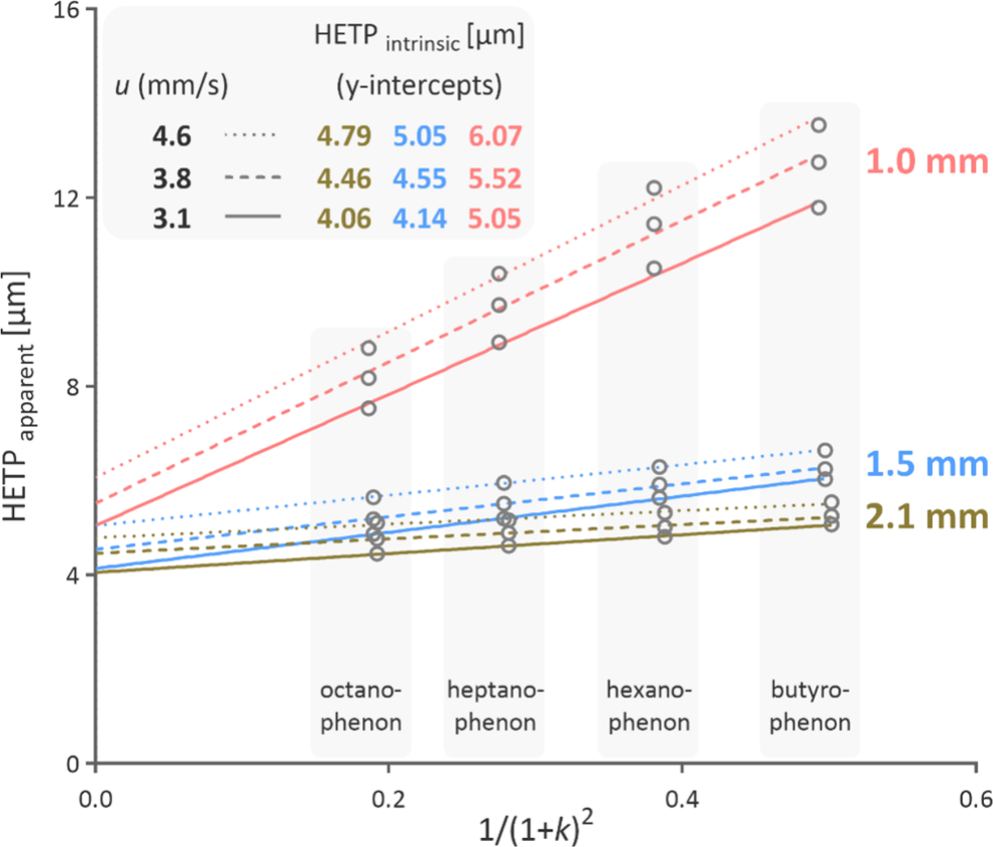
Intrinsic height equivalents of theoretical plates (HETP) for columns with 1.0, 1.5, and 2.1 mm inner diameters at linear velocities (*u*) near optima. HETP values were determined using a homologous alkylphenone series, following the method described by Gritti and Guiochon.^18^ The intrinsic HETP corresponds to the *y*-intercept in plots of 1/(1 + *k*)^2^ against observed HETP, where *k* is the retention factor.

Based on the findings of Gritti, we first calculated the theoretical distribution of the bulk and intermediate zones in the three selected columns packed with 2.7 μm particles. For the 2.1 mm i.d. column, the bulk zone extended across 76% of the entire column diameter, indicating the least heterogeneous bed structure among the three columns. In contrast, the 1.0 mm i.d. column exhibited the least favorable intermediate-to-bulk ratio, with the intermediate zone occupying about 45% of the entire column diameter, resulting in the worst packing uniformity. The 1.5 mm i.d. column demonstrated better packing homogeneity compared to the 1.0 mm i.d. column, with the intermediate zone accounting for only 32% of the total column volume (Figure 1). The 1.5 mm i.d. column is, therefore, likely to have reduced trans-column eddy dispersion compared to the 1.0 mm i.d. column due to its improved bed uniformity, though still not as optimized as the 2.1 mm i.d. column. The calculations aligned with the true column efficiencies that we determined at three near-optimum flow rates where the eddy dispersion most significantly contributes to the in-column band broadening. To eliminate the contribution of the extra-column dispersion to the observed peaks, we followed the procedure based on a series of homologous alkylphenons developed by Gritti and Guiochon (Figure 2).^18^

The flow rates were set based on column cross sections and were not adjusted to linear velocities of the mobile phase necessary for purely chromatographic column evaluation. Such adjustments would lead to unequal gradient times between the columns, which could compromise unbiased proteomic evaluation due to differences in the total time for acquiring DDA data. We believe that any differences in linear velocities between columns were minimal, and their impact on proteomic data was much less significant than differences in the total MS spectra acquisition time.

### LC-MS Analysis of iRT Peptides

First, we assessed the chromatographic performance of columns with 2.1, 1.5, and 1.0 mm i.d. using a mixture of 11 well-characterized iRT peptides developed to normalize the retention time of other peptides.^17^ Regrettably, the intensities for the last two eluting peptides gradually decreased with time. This problem was likely caused by the nonspecific adsorption of hydrophobic peptides on the surface of the glass vial; however, attempts to resolve this by analyzing the samples immediately after preparation or adding PEG 20,000 to the sample were unsuccessful.^20^ As a result, these peptides were not evaluated.

**Figure 3:**
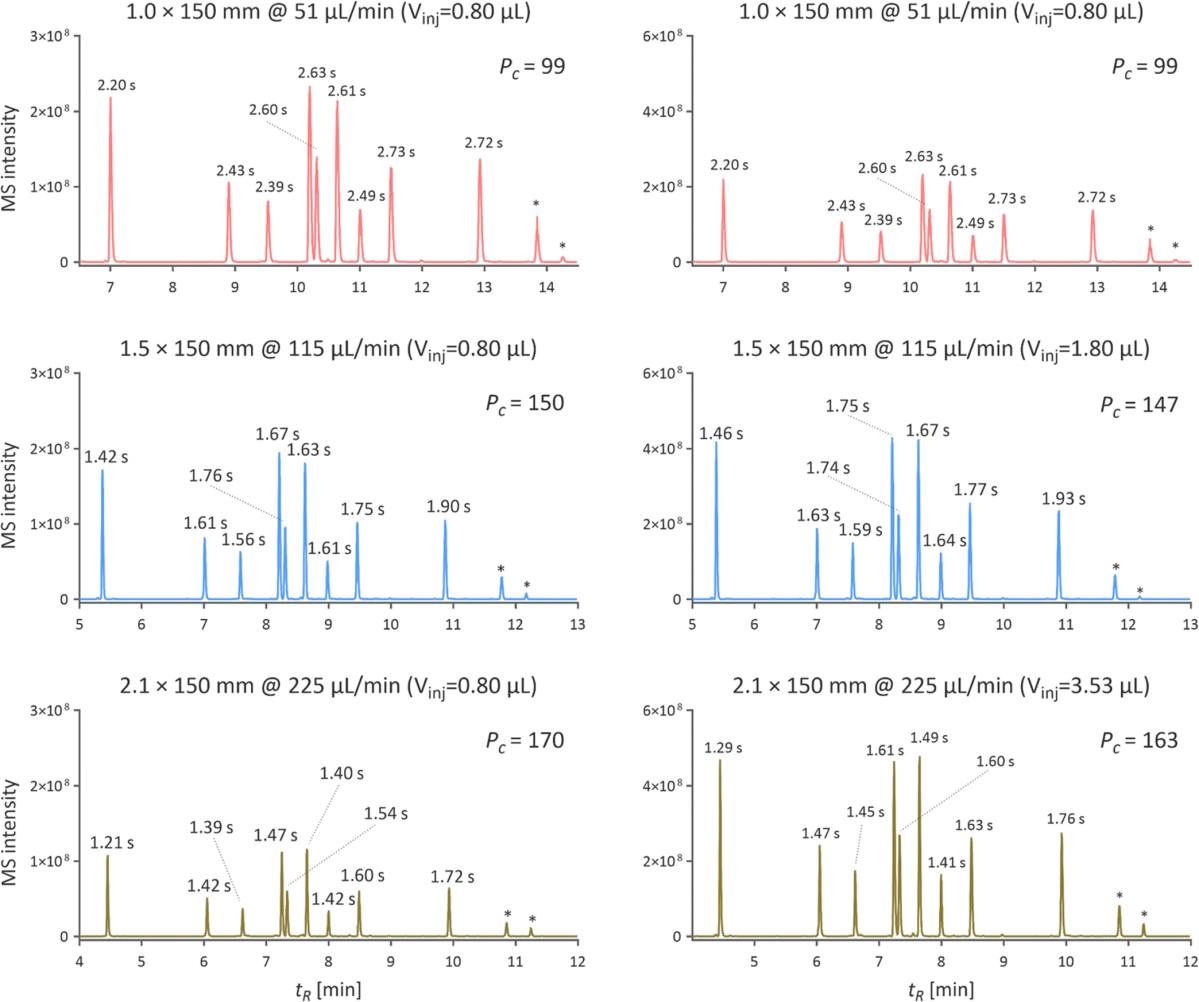
Chromatograms from the separation of constant (left) and proportional (right) amounts of iRT peptides using columns with different internal diameters. Peak capacity (*P*_c_) was calculated considering the elution window being 7 min. The chromatogram from the 1.0 mm i.d. column for the proportional injection is the same as on the left, but the MS intensity range was adjusted. The peak widths *w*_h_ in seconds are shown above each peak. The peaks with * were not evaluated because of their continuous decline in intensity.

For each column, we separated 0.8 μL of iRT peptides of approximately one pmol/μL concentration using a 12 min linear gradient running from 2.5 to 52.5% component B. The 2.1 mm i.d. column produced the narrowest peaks at a constant injection volume with an average *w*_h_ of 1.46 s (Figure 3), consistent with the recently published literature.^15^ The 1.5 mm i.d. column showed only 13% higher average *w*_h_ compared to the 2.1 mm i.d. column, whereas the 1.0 mm i.d. column separated iRT peptides with average *w*_h_ higher by 74% than the 2.1 mm i.d. column, confirming that the chromatography of 1.5 mm i.d. column is much closer to that of the 2.1 mm i.d. column. Given the minimized postcolumn band dispersion by using very narrow capillaries with integrated fittings, these findings accentuated that the trans-column eddy dispersion caused by the radial flow heterogeneity reduced the efficiency of 1.0 mm i.d. columns, making them suboptimal for high-flow proteomics, particularly if used on LC systems with the extra-column band dispersion not completely minimized.^4^

At constant injection volume, the MS intensities should have theoretically decreased by 56 and 77% for 1.5 mm i.d. and 2.1 mm i.d. against the 1.0 mm i.d. column. However, because of the broader peaks observed with the 1.0 mm i.d. column, the actual decline was only 23% for the 1.5 mm i.d. column and 53% for the 2.1 mm i.d. columns. To further investigate if this difference is solely due to broader peaks, we injected the iRT peptides in volumes proportional to column cross sections: 0.8, 1.8, and 3.53 μL for 1.0, 1.5, and 2.1 mm i.d. columns. Under such conditions, the peak area should be theoretically constant if there is no difference in ESI efficiency. Nevertheless, the peak areas increased by 18 and 30% for 1.5 and 2.1 mm i.d. columns, compared to the 1.0 mm i.d. column, despite the ESI conditions set according to the mobile phase flow rate. The findings suggest the ESI settings must be systematically adjusted when using lower i.d. columns, while columns with larger i.d. are more robust in this regard.

Overall, the results concluded that the 1.5 mm i.d. column ideally balances the high sensitivity offered by a 1.0 mm i.d. column and the superior separation efficiency of a 2.1 mm i.d. column for separating peptides. The sensitivity provided by the 2.1 mm i.d. column was far behind the one offered by the 1.0 mm i.d. column and, hence, was not further evaluated for its applicability in proteomics analysis.

### LC-MS/MS Analysis of Trastuzumab Tryptic Peptides

**Figure 4:**
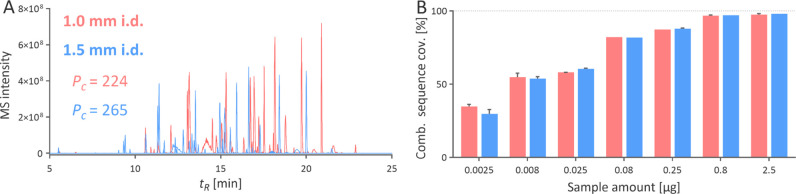
Extracted ion chromatograms of 0.8 μg of trastuzumab tryptic peptides separated using 1.0 and 1.5 mm i.d. columns (A). The peak capacity (*P*_c_) was calculated considering the 140 peptides identified using both columns. Combined sequence coverage for trastuzumab when various amount of its tryptic digest was analyzed using 1.0 and 1.5 mm i.d. columns (B).

Inspired by the promising results obtained using a 1.5 mm i.d. column to separate standard peptides, we sought to evaluate the column performance in terms of protein coverage by analyzing the tryptic digest of trastuzumab and comparing the results with the 1.0 mm i.d. column. Near half-log diluted amounts of trastuzumab tryptic peptides ranging from 2.5 ng to 2.5 μg were separated on both columns using a 22 min linear gradient from 2 to 52% component B. Despite the improved detection sensitivity offered by the 1.0 mm i.d. column, the 1.5 mm i.d. column provided a comparable protein coverage across all sample amounts (Figures 4 and S1). The only exception was the lowest sample amount of 2.5 ng, where the 1.0 mm i.d. column provided slightly better sequence coverage. The 1.5 mm i.d. column displayed superior performance in terms of *w*_*h*_ and capacity compared to the 1.0 mm i.d. column. This improved chromatographic performance likely balanced the high sensitivity of 1.0 mm i.d. column, resulting in similar protein coverage. Our results confirmed that the 1.5 mm i.d. format is very suitable for peptide mapping of protein biopharmaceuticals.^16^

### Analysis of Tryptic Peptides of *F. tularensis* LVS Proteins

The quality of the proteomics methods is usually judged by the depth of protein and peptide identification they provide. Also, the samples in bottom-up proteomic analyses are generally of high complexity and do not consist of just one protein. Given these considerations, we evaluated the performance of the 1.5 mm i.d. column on a more complex sample. A serial dilution of tryptic digest from the *F. tularensis* LVS (from 8 ng to 25 μg) was analyzed using columns with 1.0 and 1.5 mm i.d using a 30 min linear gradient from 2 to 50% component B. The goal was to confirm whether the improved chromatographic performance of the 1.5 mm i.d. column could compensate for the higher sensitivity of the 1.0 mm i.d. column, potentially resulting in a comparable or higher extent of identification.

**Figure 5:**
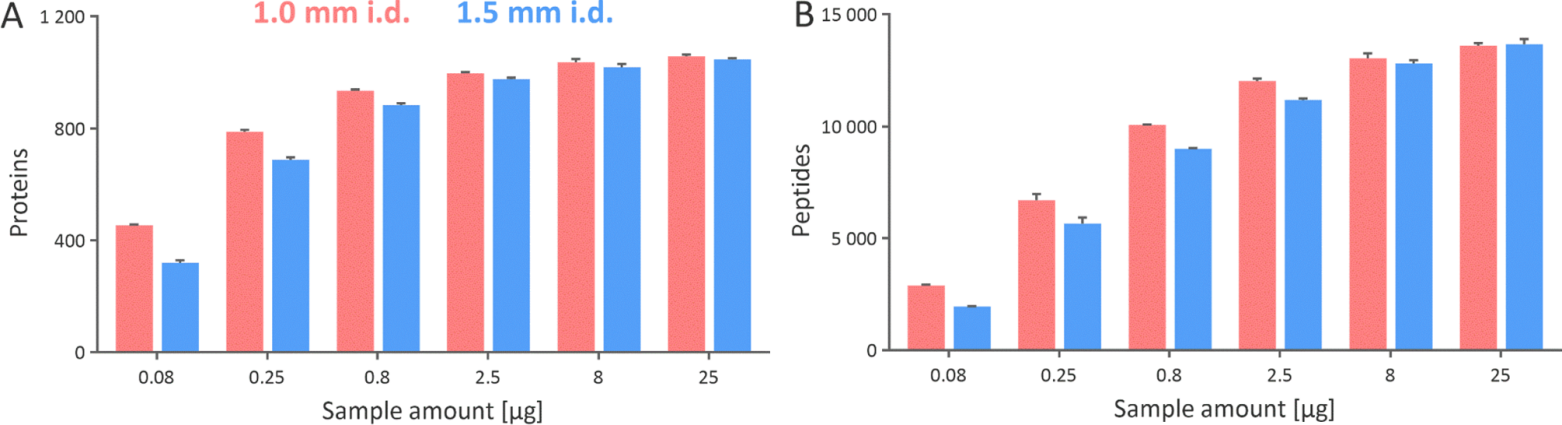
Numbers of identified proteins (A) and peptides (B) from the tryptic digest of *F. tularensis* when using columns with inner diameters of 1.0 mm (red) and 1.5 mm (blue).

Naturally, the number of identified proteins and peptides increased with the sample amount (Figure 5). The 1.5 mm i.d column achieved comparable identifications to the 1.0 mm i.d. column at higher injected sample amounts. However, the 1.0 mm i.d column at lower sample amounts provided higher numbers of identified peptides and proteins. The additional peptides identified exclusively using a 1.0 mm i.d. column had lower median intensity than those identified using both columns (median of 3.2 × 10^5^ for additional unique peptides versus a median of 8.3 × 10^5^ for common peptides, Figure S2). This suggests that the additional peptides identified are relatively low in intensity and did not cross the MS1 intensity threshold for triggering a DDA scan when using the 1.5 mm i.d. column. However, at a higher sample load, where most of the peptide intensities cross the set intensity threshold, the improved MS sensitivity of 1.0 mm i.d. column does not translate to additional peptide identification. This concludes that the superior separation performance of the 1.5 mm i.d. column offers comparable protein and peptide identification to the 1.0 mm i.d. column at higher sample amounts.

### Analysis of Real-Life Samples of Jurkat Cells at Different Gradient Lengths

In bottom-up proteomics, researchers typically tailor the LC gradients to the sample complexity, separation efficiency, speed, and throughput. To this end, we evaluated the performance of 1.5 mm i.d. and 1.0 mm i.d. columns across 15, 30, and 60 min gradient lengths using a serial dilution of Jurkat cell digest (from 50 ng to 50 μg). Peptides were separated using a gradient of 2 to 60% component B.

**Figure 6:**
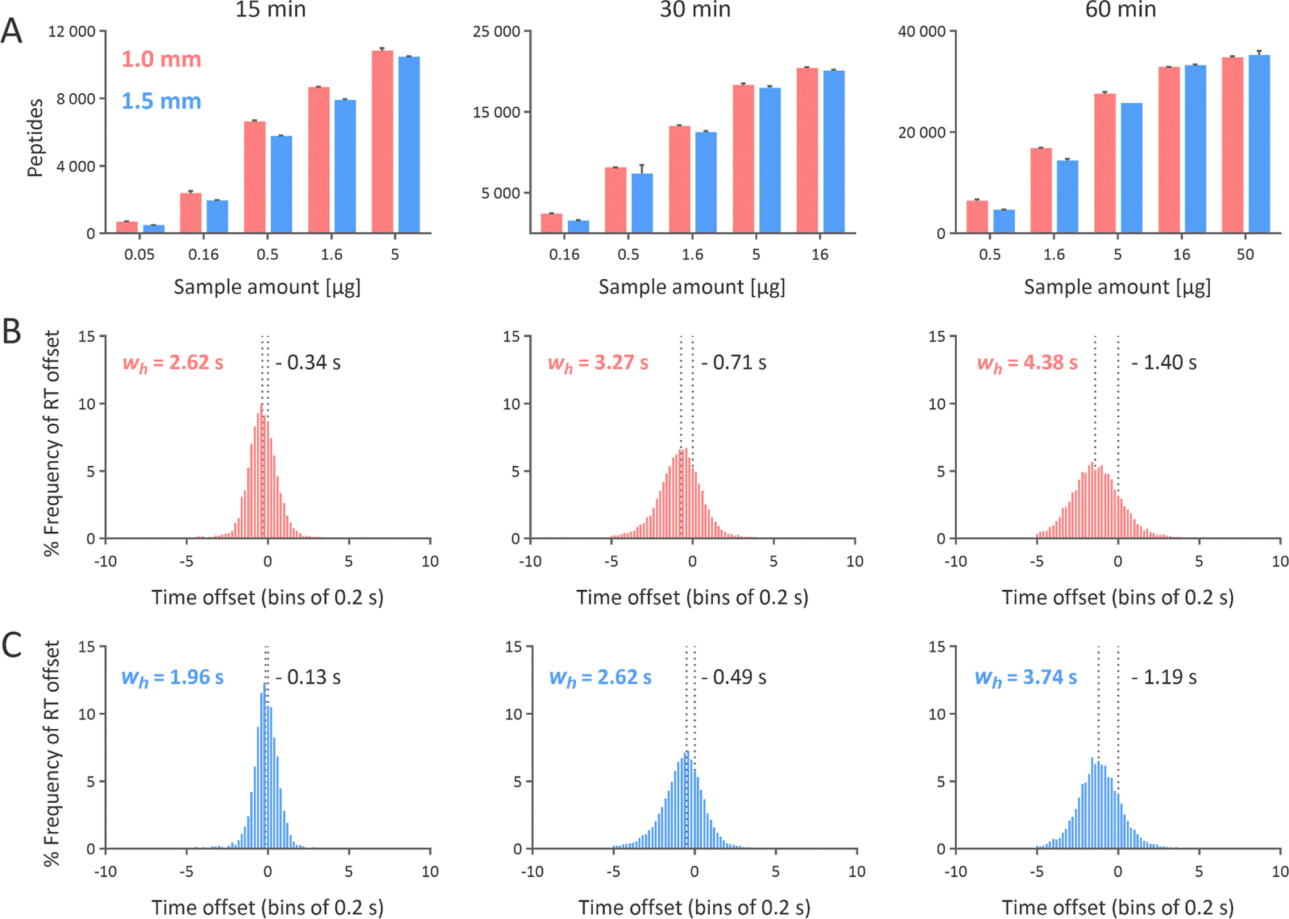
Serial dilution experiment conducted using 15, 30, and 60 min gradient lengths for 1.0 mm i.d. and 1.5 mm i.d. columns: the number of identified peptides (A), frequency distribution of the offset between the peak apex and the time when MS2 scan was recorded when measured using the 1.0 mm i.d. column (B) and 1.5 mm i.d. column (C) for 5 μg of Jurkat tryptic peptides.

**Figure 7:**
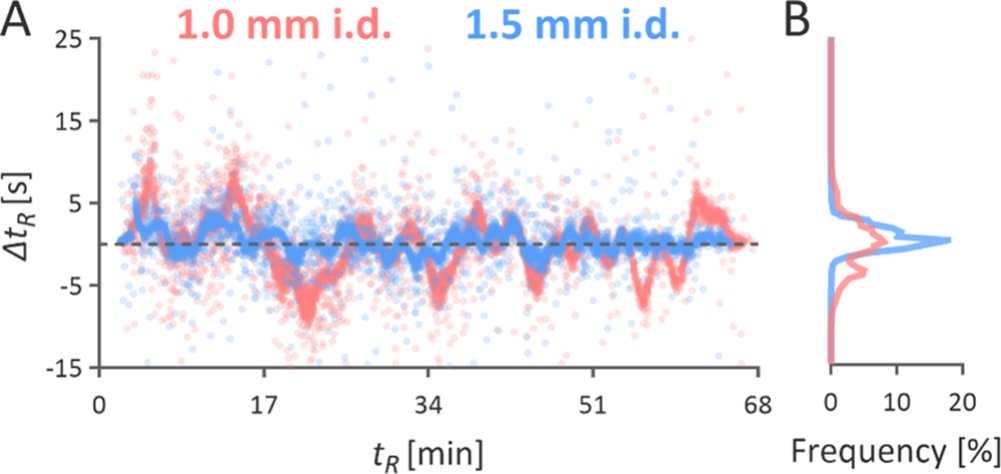
Retention time variation in seconds (A) and distribution of peptides based on their retention time variation (B) between duplicate injections of 50 μg of Jurkat tryptic peptides separated using a 60 min gradient.

At lower sample amounts, the 1.0 mm i.d. column provided greater identification of peptides (Figures 6A and S3A). However, at higher sample amounts, the 1.5 mm i.d. column provided comparable identifications to the 1.0 mm i.d. column. Linear regression revealed a strong dependency (*R*^2^ ≥ 0.98) between the injected sample amount and the summed peptide intensity (Figure S4), confirming no column or detector saturation. The median peptide Byonic scores were comparable for both columns for all sample amounts injected (Figure S3B), further supporting comparable qualitative performance. Relative spectral quality calculated as PSMs/recorded MS2 spectra × 100 was slightly better for the 1.0 mm i.d. column at lower sample amounts. However, both columns approached comparable values at higher sample amounts (Figure S3C). The 1.5 mm i.d. column displayed lower identification redundancy calculated as a ratio of PSMs to identified peptides (Figure S3D), which is typical for high-quality peptide separation.^4^ Narrower peaks trigger only one DDA scan of a precursor closer to the peak apex, enhancing the quality of fragmentation spectra.^1^ Indeed, a detailed data evaluation confirmed that the narrower peaks obtained using the 1.5 mm i.d. column triggered the DDA scans closer to the precursor peak apex than the peaks obtained using the 1.0 mm i.d. column (Figure 6B,C). The 1.5 mm i.d. column displayed a more stable chromatographic performance than the 1.0 mm i.d. column (Figure 7).

### Robustness and Repeatability of Analyses Using the 1.5 mm i.d. Column

**Figure 8:**
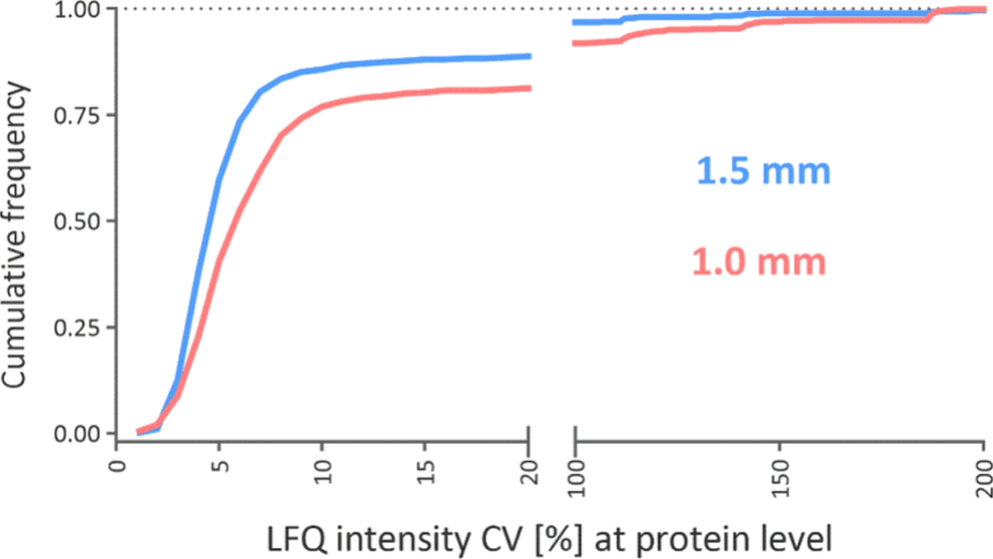
Cumulative frequency distribution of protein quantification reproducibility between nine replicates of Jurkat cell digest.

To further demonstrate the robustness and quantitative ability of the 1.5 mm i.d. column, we performed an experiment consisting of nine replicate injections of 500 ng of Jurkat cell digest, separated using a gradient of 2 to 40% component B in the mobile phase in 30 min over 3 days using 1.0 and 1.5 mm i.d. columns. The retention time of identified peptides showed a median CV of 0.002% for the 1.0 mm i.d column and 0.001% for the 1.5 mm i.d. column (Figure S5). The stable chromatographic performance of the 1.5 mm i.d column compared to the 1.0 mm i.d. column also led to a higher reproducibility of protein quantification (Figure 8), which is, nonetheless, very good already using the 1.0 i.d. columns.^4,21^ The median % CV for the quantified protein was 5.0% for the 1.5 mm i.d. column and 6.2% for the 1.0 mm i.d. column.

The results demonstrate that the better peptide chromatography of the 1.5 mm i.d. column can balance the lower signal response, providing comparable identification as the 1.0 mm i.d. column. Still, some strategies can be used to improve the MS sensitivity using a 1.5 mm i.d. column, such as the addition of additives such as DMSO^22^ or ethylene glycol in the mobile phase,^23^ the use of acetic acid as a mobile phase acidifier,^24^ modification of desolvation gas,^25^ etc. Fragmentation of precursors closer to the peak apex will significantly reduce cofragmentation and provide a better signal-to-noise ratio.^26^ This will also lead to improved quantitative accuracy along with proven reproducibility.

## Conclusions

As the adoption of microflow LC-MS/MS configuration continues to grow, our study demonstrates the 1.5 mm i.d. column as a compelling alternative to the currently used 1.0 mm i.d. columns. Overall, the 1.0 mm i.d. column offers a slight advantage in peptide identification at a lower sample amount. However, the superior chromatographic performance of the 1.5 mm i.d. column compensates for the lower MS intensity and provides comparable peptide and protein identification when sample amounts are sufficient, which is typically achievable when using a microflow setup for proteomic workflow. The 1.5 mm i.d. column offers the added benefits of enhanced chromatographic stability. These 1.5 mm i.d. columns show promise for microflow 2D-LC/MS proteomic analyses, where the higher flow rates implied when using such columns can minimize the overdue times in the second dimension. Our future work will explore this application of the 1.5 mm i.d. columns. Unfortunately, not many manufacturers produce columns with such atypical inner diameter, but we anticipate that will change with time. Encouragingly, despite their relatively limited availability, the cost of these columns does not differ significantly from those with 1.0 or 2.1 mm i.d.

## Supporting Information Available

Sample preparation (Note S1); data evaluation (Note S2); HESI-II parameters for individual flow rates (Table S1); MS1 and DDA settings for all experiments (Table S2); summed MS peptide intensity of trastuzumab peptides (Figure S1); peptides of *F. tularensis* LVS identified in 1 × 10^4^ intensity bins during a 30 min analysis (Figure S2); serial dilution experiments at 15, 30, and 60 min gradient length for 1.0 mm i.d. and 1.5 mm i.d. column (Figure S3); linear regression plots of the summed peptide intensity (Figure S4); frequency distribution of peptide retention times (Figure S5) (PDF)
