# Supplementary material for: Microflow LC-MS Bottom-Up Proteomics Using 1.5 mm Internal Diameter Columns
Source: ACS Omega. 2025 Jan 24;10(4):4094–101. doi: 10.1021/acsomega.4c10591 (PMC11800007; doi:10.1021/acsomega.4c10591)

Supporting Information

Microflow LC-MS bottom-up proteomics using 1.5 mm internal diameter columns

Siddharth Jadeja [1], Denis K. Naplekov [1], Mykyta R. Starovoit [1], Kateřina Plachká [1], Harald Ritchie[2], Jason Lawhorn[2], Hana Sklenářová [1], Juraj Lenčo [1*]

[1] Department of Analytical Chemistry, Faculty of Pharmacy in Hradec Králové, Charles University, Heyrovského 1203/8, 500 05 Hradec Králové, Czech Republic

[2] Advanced Materials Technology, 3521 Silverside Road, Suite 1-K, DE 19810, Wilmington, United States

[*]Corresponding Author: E-mail: lenco@faf.cuni.cz, Phone: +420 495 067 381.

Contents

Note S1. Sample preparation 2

Note S2. Data evaluation 3

Table S1. HESI-II parameters for individual flow rates 4

Table S2. MS1 and DDA settings for all experiments 5

Figure S1. Summed MS peptide intensity of 140 trastuzumab peptides 6

Figure S2. Peptides identified in $1e^4$ intensity bins during a 30-minute analysis of the *F. tularensis* LVS digest. 7

Figure S3. Serial dilution experiments at 15, 30, and 60 min gradient length for 1.0 mm i.d. and 1.5 mm i.d. column 8

Figure S4. Linear regression plots of summed peptide intensity 9

Figure S5. Frequency distribution of peptide retention times 10

Note S1. Sample preparation

Digestion of trastuzumab

The tryptic digest of trastuzumab was prepared using a SMART Digest™ kit (Thermo Fisher Scientific). The reaction mixture with 100 µg of trastuzumab was composited according to the manufacturer's instructions and digested in a thermomixer at 70 °C and 1400 rpm for 45 min. The peptides were reduced in 5 mM tris(2-carboxyethyl)phosphine hydrochloride at 37 °C for 60 min. Thiols were thiomethylated at 22 °C for 60 min after adding 1.13 µL of 4M S-methyl methanethiosulfonate. The mixture was acidified with 2 µL of 10% trifluoroacetic acid and filtered through a 0.22 µm spin filter (Corning). The peptides were desalted and diluted with 0.1% TFA to a final concentration of 1 µg/µl.

Trypsin digestion of *Francisella tularensis* LVS cells proteins

The *Francisella tularensis* LVS pellet, obtained from Chamberlain medium culture with OD_{600} of 0.6 to 0.7, was washed and lysed in 2% sodium deoxycholate at 70 °C. One milligram of proteins was buffered with Tris–HCl, pH 7.5 (Serva), and incubated with 250 U of benzonase. Proteins were reduced in 20 mmol/L dithiothreitol, and thiols were blocked using 50 mmol/L chloroacetamide. Proteomics-grade trypsin (SOLu-Trypsin) was added in a 1:50 ratio, and the proteins were digested at 37 °C overnight. The digests were acidified with TFA to quench the enzymatic reaction and induce precipitation of deoxycholic acid, which was subsequently extracted into ethyl acetate saturated with water. The remnants of ethyl acetate were evaporated in a vacuum centrifuge at 30 °C within 30 min. The peptides were desalted and diluted with 0.1% TFA to a final concentration of 1 µg/µl.

Trypsin digestion of Jurkat cells proteins

The Jurkat cells (ATCC TIB-152) were cultivated in RPMI 1640 medium supplemented with 10% fetal bovine serum. After a wash with phosphate-buffered saline, cells were lysed in 2.5% sodium deoxycholate containing 125 U/mL benzonase. The protein concentration was determined using a bicinchoninic acid assay. One milligram of proteins was digested using a similar procedure as specified for the *F. tularensis* LVS and reconstituted to 1 µg/µL concentration in 0.1% TFA.

Desalting of tryptic peptides

Tryptic peptides in both digests were desalted using Pierce Peptide Desalting Spin Columns (Thermo Fisher Scientific). The peptides were eluted using 50% acetonitrile acidified with 0.1% TFA and vacuum dried. The peptides from all the tryptic digests were redissolved in 0.1% aqueous TFA to the required concentration.

Note S2. Data evaluation

The LC-MS data for trastuzumab were searched against the FASTA sequence of trastuzumab downloaded from the DrugBank using Byonic v3.5. A semispecific tryptic cleavage with a maximum of one missed cleavage allowed was used. The mass tolerance was set at 7 ppm for precursors and 17 ppm for fragments. Thiomethylation of Cys was set as a fixed modification, while oxidation of Met and formation of pyroGlu from Glu and Gln was set as a dynamic modification. The peptides were screened for 57 *N*-glycans, typical for human plasma proteins. Spectra of identified peptides and their retention times were stored in the Skyline library, where the peak properties of identified peptides were evaluated.

The LC-MS data for *F. tularensis* LVS and Jurkat cells were searched in Proteome Discoverer v2.3 using integrated Byonic v3.5.0 (Protein Metrics). After data recalibration, the spectra were searched against the *F. tularensis* (UP000076142, downloaded in July 2021) human FASTA protein database (UP000005640, downloaded in May 2020) downloaded from the UniProt. A semispecific tryptic cleavage with a maximum of one missed cleavage was allowed. The mass tolerance was set at 7.5 ppm for precursors and 18 ppm for fragments. Carbamidomethylation of Cys was set as a fixed modification. Oxidized Met, pyroGlu formation from N-terminal Glu and Gln, and deamidation of Asn were set as dynamic modifications. Acetylation of protein at the N-terminal was an additional dynamic modification selected for analysis of Jurkat cell data. Peptide-spectrum matches (PSMs) identified with the 2D FDR ≤ 1.0 % were considered.

The LC-MS data Jurkat cells for quantitative reproducibility analysis were searched using the Andromeda search engine in MaxQuant v 2.0.1.0 using the FASTA database as described above. The search parameters were set to carbamidomethylation of cysteine as fixed modifications and methionine oxidation, cyclization of N-terminal glutamate and glutamine, N-terminal acetylation, and deamidation of Asn as variable modifications. The mass spectra were recalibrated with a peptide tolerance of 20 ppm first. The main search was carried out with a mass tolerance of 4.5 ppm. Fragment ion mass tolerance was set to 20 ppm. The results were filtered with a false discovery rate of 0.01, and a match between the runs and label-free quantification was enabled.

Table S1. HESI-II parameters for individual flow rates recommended by the controlling software

	Flow rate:	51 µL/min	115 µL/min	225 µL/min
Parameter:				
Sheath gas flow rate (A. U.)		30	37	46
Auxiliary gas flow rate (A. U.)		10	10	10
Sweep gas flow rate (A. U.)		1	1	2
Capillary temperature (°C)		250	250	252
Auxiliary gas temperature (°C)		151	230	403
Depth of the ESI needle		halfway between B-C for all flow rates		

Table S2. MS1 and DDA settings for all experiments

**Analysis of iRT peptides**		
MS1 settings	Resolution at 200 m/z	60,000
	AGC target	1×10^6
	Maximum injection time	60 ms
	Scan range	350 to 1500 m/z
**Analysis of trastuzumab digest**		
MS1 settings	Resolution at 200 m/z	60,000
	AGC target	1×10^6
	Maximum injection time	110 ms
	Scan range	300 to 2000 m/z
DDA and MS2 settings	Charge states of precursors	≥2 and ≤5
	Isolation window	3 m/z
	Normalized collision energy for HCD	27
	Resolution at 200 m/z	15 000
	AGC target	2×10^5
	Maximum injection time	120 ms
	Exclusion time	3 s
	Max. number of precursors	2
**Analysis of *F. tularensis* LVS digest and Jurkat cell digest**		
MS1 settings	Resolution at 200 m/z	60,000
	AGC target	3×10^6
	Maximum ion time	110 ms
	Scan range	350 to 1500 m/z
DDA and MS2 settings	Charge states of precursors	≥2 and ≤5
	Isolation window	1.8 m/z
	Normalized collision energy for HCD	27
	Resolution at 200 m/z	15 000
	AGC target	2×10^5
	Maximum injection time	50 ms
	Exclusion time	20 s
	Max. number of precursors	10

Figure S1. Summed MS peptide intensity of 140 trastuzumab peptides commonly identified using both columns (A). The peak width (w_h) for the identified peptides when separated using 1.0 (red) and 1.5 mm (blue) i.d. columns with the line and the numeric value representing mean (B).

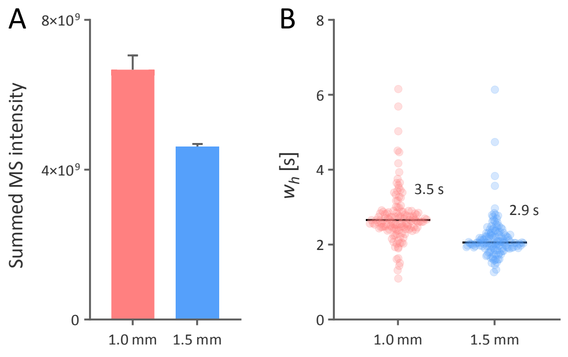

Figure S2. Peptides identified in 1e^4 intensity bins during a 30-minute analysis of the *F. tularensis* LVS digest.

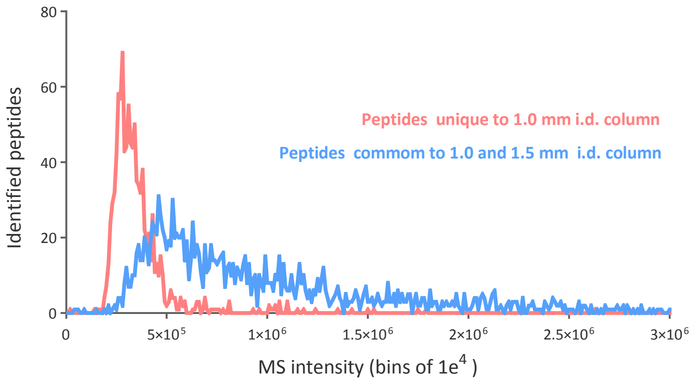

Figure S3. Serial dilution experiments at 15, 30, and 60 min gradient length for 1.0 mm i.d. and 1.5 mm i.d. column. The identification of protein groups (A). Boxplots of Byonic score for the identified peptides (B). Percentage of PSM numbers to the identified MS/MS spectra for both columns at different injected amounts using three different gradient lengths (C). The ratio of PSM numbers to the number of peptides identified for both columns at different injected amounts using three different gradient lengths (D).

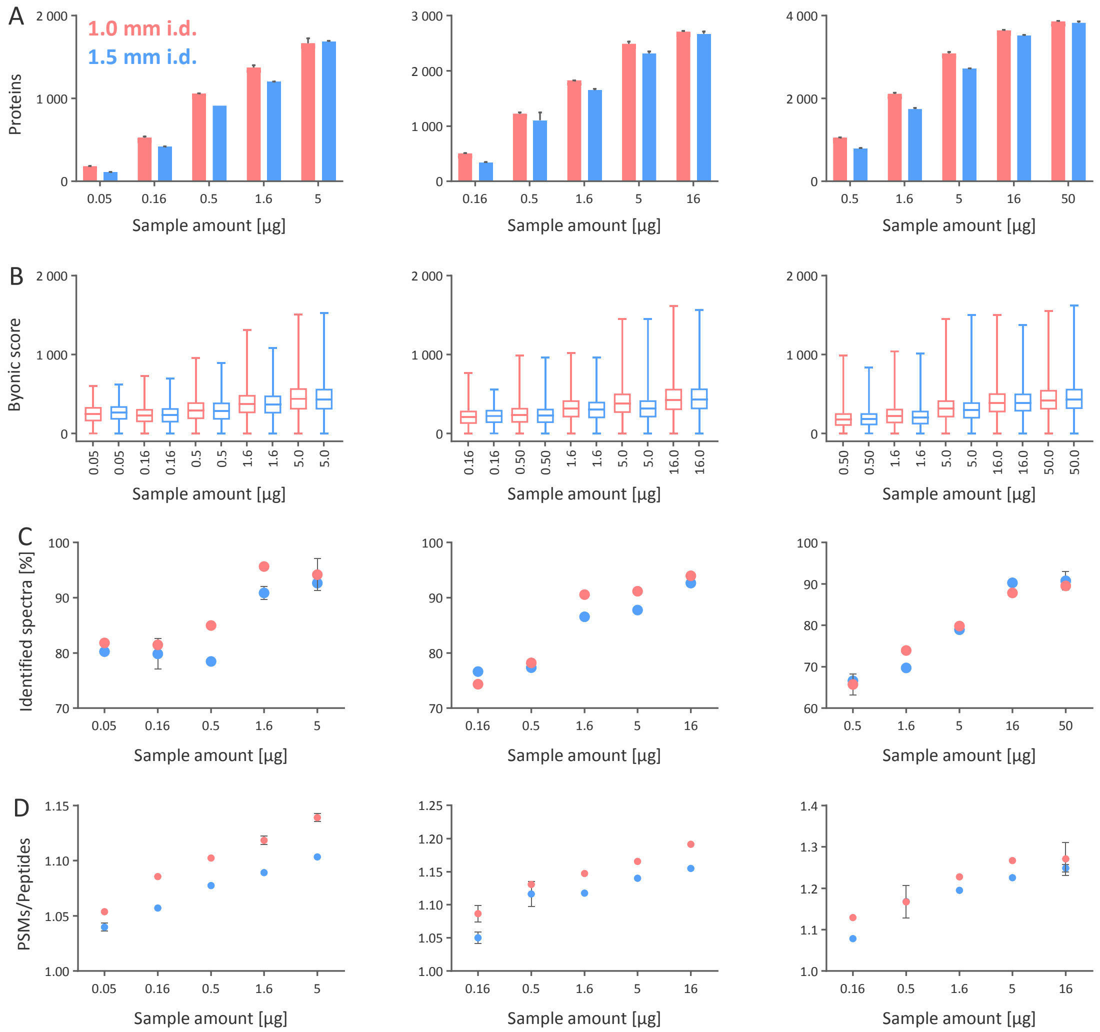

Figure S4. Linear regression plots of summed peptide intensity against sample amount observed in analyses using 1.0 mm i.d. (A) and 1.5 mm i.d. (B) columns

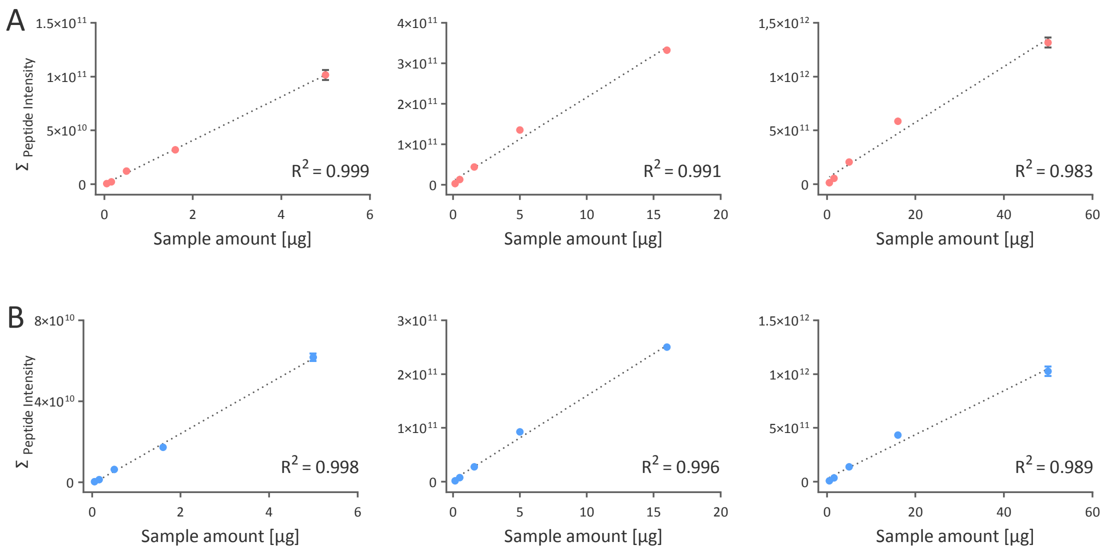

Figure S5. Frequency distribution of peptide retention times (t_R) of Jurkat cells between nine replicates

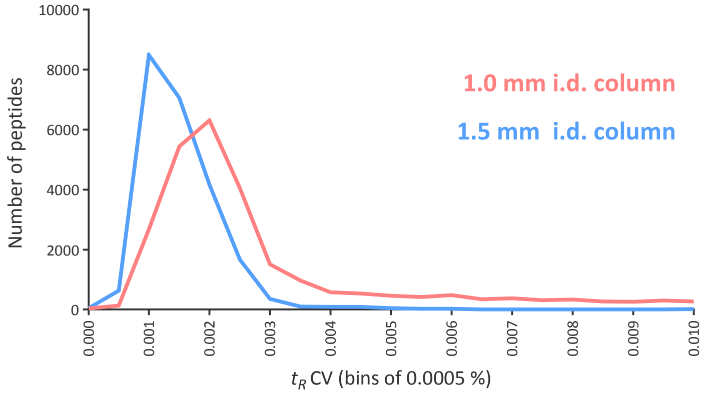

Supplement: Supplementary file 1 — ao4c10591_si_001.pdf [file ao4c10591_si_001.pdf]
